# Status of Body Contouring Following Metabolic Bariatric Surgery in a Tertiary Hospital of Greece—Still a Long Way to Go

**DOI:** 10.3390/jcm12093196

**Published:** 2023-04-29

**Authors:** Athanasios G. Pantelis, Georgios Vakis, Maria Kotrotsiou, Dimitris P. Lapatsanis

**Affiliations:** 1 Bariatric and Metabolic Surgery Unit, 4th Department of General Surgery, Evaggelismos General Hospital of Athens, Ipsilantou 45-47, 106 76 Athens, Greece; dimitrislapatsanis@gmail.com; 2 Department of Plastic and Reconstructive Surgery, Evaggelismos General Hospital of Athens, Ipsilantou 45-47, 106 76 Athens, Greece; g_vakis@hotmail.com (G.V.); markotro@otenet.gr (M.K.)

**Keywords:** body contouring, bariatric surgery, metabolic surgery, abdominoplasty, breast contouring, thigh contouring, arm contouring

## Abstract

Obesity is a disease rather than a state, and metabolic bariatric surgery (MBS) is its most effective treatment. Body contouring surgery (BCS) is an integral part of the continuum of care following MBS, provided that the body mass index (BMI) has stabilized for an adequate period. This study is an attempt to capture the current status of BCS following MBS in Greece, based on data from one of the country’s highest-volume hospitals. We recruited patients from the Bariatric and Plastic-Reconstructive Surgery registries who had undergone both MBS and BCS and invited them to answer a structured questionnaire with components on demographics, safety and effectiveness of previous operations, quality of life (QoL), body image, social activity, sexual activity, and doctor–patient communication. Twenty-four patients participated in the survey (response rate 88.1%). The mean BMI pre-MBS was 43.8 kg/m^2^ and that pre-BCS was 28.6 kg/m^2^. Based on the Bariatric sub-cohort, only 2.5% of post-bariatric patients underwent BCS. The mean interval between MBS and BCS was 2.9 years. The distribution of patients by MBS was as follows: sleeve gastrectomy 8 (33.3%), gastric band 7 (29.2%), gastric bypass 5 (20.8%), and gastric plication 2 (8.3%). The distribution of patients by BCS was as follows: abdominoplasty 23 (94.7%), breast contouring 8 (33.3%), thigh contouring 3 (12.5%), and arm contouring 5 (20.8%). Most positive components (70.6%) regarding QoL were appraised by >80% of the participants, indicating overall satisfaction after BCS. Conversely, only 12.5% of negative components were endorsed by >20% of patients. In conclusion, BCS has a low prevalence after MBS, although it is related to an improved quality of life and body image.

## 1. Introduction

It is widely acceptable that metabolic bariatric surgery (MBS) is the most effective, safe, and sustainable way of losing weight and attenuating the impact of obesity-associated medical problems, including but not limited to hypertension, type 2 diabetes mellitus, dyslipidemia, obstructive sleep apnea, etc., according to pertinent evidence [1,2,3,4]. According to current knowledge, obesity is considered a disease [5,6]; as such, MBS is not the final step, but rather a pivotal component in the continuum of obesity multimodality care. The management of obesity is multidisciplinary, as mandated by the recently released ASMBS-IFSO joint statement on the indications for metabolic and bariatric surgery [7], and it is within this context that body contouring surgery following MBS is a field of rigorous scientific focus.

Body contouring is considered an aesthetic intervention or an unnecessary luxury rather than an integral part of the well-being and the quality of life of the post-bariatric patient, and this holds true for patients, policymakers, insurance companies, and even non-familiar healthcare providers alike. In a large registry-based study of more than 37,000 post-MBS patients, only 5.58% among them had a subsequent body contouring surgery (BCS) [8], but according to other sources, there is great and multifactorial variability within this figure (6–40%) [9]. Contrary to common belief, post-bariatric BCS may contribute to further and sustainable weight loss. In a recent meta-analysis of 11 comparative studies, ElAbd et al. found that patients who underwent BCS had a statistically significant increase in their percentile total weight loss (%TWL), by 6%, and in their percentile excess weight loss (%EWL), by 14% [10]. What are the underlying mechanisms for this effect? Beyond the simplistic approach that BCS reduces the total mass of adipose tissue, the additional impacts of BCS on the ratio of white (WAT) and brown adipose tissue (BAT), distribution (subcutaneous vs. visceral) of WAT according to phenotype (g vs. android), alteration of function of adipose tissue macrophages (ATMs), and their role in endoplasmatic reticulum stress, oxidative stress, and inflammation, which are inseparably linked with obesity, remain elusive from a pathophysiologic point of view [11,12,13,14].

Quality of life is a metric that was neglected in the past but is taken into serious account with increasing frequency in the bariatric literature. Current evidence suggests that BCS contributes substantially to the amelioration of the global quality of life of the bariatric patient, including improvements of the individual indices: physical, psychological, and social functioning, body image, sexual function, self-esteem, reduction of pain, and personal hygiene [15,16,17]. Most importantly, specific tools that quantify the impact of BCS on QoL have been developed [18], thus enabling comparisons between subpopulations and allowing for the further individualization of therapy.

Despite the evidence on the benefits, there is no consensus on the ideal timing for performing BCS after MBS. Most guidelines converge in that BCS should be attempted at least 12–18 months after MBS, with the prerequisite that body weight has stabilized for at least 6–12 months, but the relevant level of evidence is low [9,19]. Yet, there is great heterogeneity among patients and surgeons alike. In addition, the role of the Dietician and/or Nutritionist is pivotal. Beyond the implementation of a healthy diet as a means of maintaining a weight plateau and bridging the temporal gap between MBS and BCS, such a specialist is an essential member of the multidisciplinary team for providing nutritional support and optimizing the nutritional status of the post-bariatric patient in view of BCS [20,21].

In light of the aforementioned documentation, with the present study, we attempted to understand trends in BCS following MBS in our facility with regards to demographic data, safety, effectiveness, and quality of life and potentially yield conclusions regarding the status of post-bariatric BCS in our country.

## 2. Materials and Methods

### 2.1. Study Design and Participants

This cross-sectional study was performed with the co-ordination of 2 hospital departments: Metabolic and Bariatric Surgery Unit (MBSU) of the 4th Surgical Department and the Department of Plastic and Reconstructive Surgery (DPRS). The study period was October–November 2022.

The study included 2 different cohorts. The one cohort consisted of patients who had undergone any MBS (including sleeve gastrectomy, Roux-en-Y or one anastomosis gastric bypass, greater curvature plication, revisional surgery for insufficient weight loss or weight regain, etc.) by the faculty of the MBSU during the 5-year period 2016–2020. With this, we wanted to ensure that at least two years had passed since the index bariatric operation. Throughout these years, there have not been any alterations in the indications for MBS, preoperative workup, surgical techniques, or postoperative follow-up protocol on behalf of the MBSU. Regarding the same disciplines, the guidelines, best practices, and indications mandated by the International Federation for the Surgery of Obesity and Metabolic Disorders (IFSO^®^) were endorsed and followed.

The other cohort comprised patients who had undergone BCS performed by the faculty of the DPRS from 2015 through November 2022. In this way, we wanted to maximize the number of participants, with the assumption that BCS has a “rare occurrence”, i.e., not many post-bariatric patients eventually end up undergoing BCS. The patients who had been submitted to BCS adhered to the relevant inclusion criteria (BMI < 30 kg/m^2^, weight stability for at least 12 months, absence of serious functional disturbances).

The patients of both cohorts were contacted by telephone or in-person in the respective outpatient clinics of each department and were invited to fill in the same participation form and answer the same questionnaire (see Section 2.2). The forms were anonymized, and the researchers had no access to any identifiable data that could potentially breach objectivity or confidentiality. For those patients who did not proceed to BCS following MBS, we recorded the reasons for not undergoing BCS but did not ask further questions. Consequently, patients from the first cohort that had undergone both MBS and BCS constituted Group 1. Apparently, all patients from the second cohort had undergone both MBS and BCS, and as such they constituted Group 2.

### 2.2. Measures

The participation form and questionnaire (File S1) consisted of the following sections: (1) Sociodemographic Data (gender, date of birth, height in meters, weight before the BCS, maximum weight as an adult, minimum weight as an adult, weight at the time of the interview, area of residency, marital status); (2) Past Medical History [obesity-associated health problems including hypertension (HTN), dyslipidemia (DLD), type 2 diabetes mellitus (T2DM), coronary artery disease (CAD)/acute myocardial infarction (AMI), congestive heart failure (CHF), stroke, chronic obstructive pulmonary disease (COPD), chronic renal failure (CRF), and additionally, vasculitis, systemic lupus erythematosus (SLE), rheumatoid arthritis (RA), inflammatory bowel disease (IBD), peptic ulcer disease (PUD), anemia, COVID-19 infection, smoking status, alcohol use, medications, and past surgical history]; (3) Safety and Efficiency [date of MBS, type of bariatric operation (adjustable gastric band, greater curvature plication, sleeve gastrectomy, Roux-en-Y gastric bypass, one-anastomosis gastric bypass, other), complications post-MBS, weight before MBS, weight 1 year after MBS, date of BCS, facility where the BCS was performed, type of BCS (abdominoplasty, breast, thigh, arms), complications post-BCS]; (4) Quality of Life (QoL), consisting of 25 YES/NO questions (Table 1). The questions were further separated into “positive” (i.e., those for which an answer of YES is compatible with a favorable QoL) and “negative” (those for which an answer of NO is compatible with a favorable QoL), and two total scores were estimated, 1 positive and 1 negative. We calculated sums, crude means, and means-by-item for each sub-questionnaire. Regarding means-by-item, we set a threshold of ≥0.80 as indicative of satisfaction for positive answers and ≥0.20 as indicative of dissatisfaction for negative answers.

### 2.3. Statistical Analysis

For statistical analysis, we utilized the GraphPad Prism online utility (GraphPad Software, San Diego, CA, USA). Given the uneven distribution of patients between the two groups and the small number of post-bariatric patients who eventually undergo BCS, we implemented nonparametric tests for the statistical analysis. Numerical variables are presented as means ± the standard deviation (SD), and their differences were evaluated with Mann-Whitney U test. Categorical variables are presented using frequencies and percentages, and their differences were evaluated with Fisher’s exact test. In all cases, a *p* value of <0.05 was considered statistically significant. For the correlation of % excess BMI loss and given answers, we tested for normality of distribution with the Shapiro-Wilk test and consequently applied logarithmic linear regression.

## 3. Results

**Figure 1:**
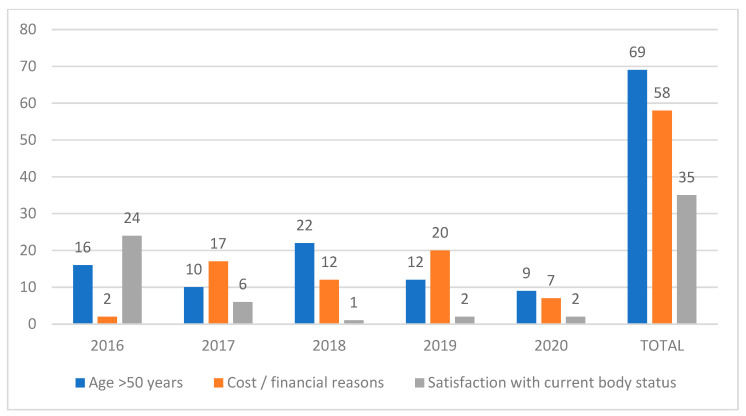
Temporal distribution of the reported reasons for which post-bariatric patients did not undergo body contouring.

The overall study cohort included 253 patients, 229 patients operated on by the MBSU and 24 patients operated on by the DPRS. The overall survey response rate was 88.1% (89.1 for MBSU patients and 79.2% for DPRS patients). Among the patients of the MBSU, only five underwent BCS (2.5%) [Group 1], within a mean interval period of 3.6 years between the last MBS and the BCS. Regarding the patients who did not undergo BCS after the MBS (N = 199), there were 37 (18.6%) who had experienced weight regain and as such were not considered eligible for BCS. Among the eligible remaining patients (N = 162), the most prevalent reason was age above 50 years (N = 69, 42.3%), followed by cost or financial reasons (58 patients, 35.8%), and satisfaction on behalf of patient of their current body status (N = 35, 21.6%). Figure 1 shows the temporal distribution of the reasons patients reported that prevented them from proceeding to body contouring following bariatric surgery.

Regarding demographic data, the mean age of both groups combined was 41.2 years, with a clear predominance of female patients (70.8%). Similarly, the majority of participants (70.8%) were residing in Athens, whereas the rest were coming from various places in Greece. Regarding marital status, there were three categories: married, single, and divorced, with married being the most frequent (66.7%). The mean BMI (body mass index) before MBS was 43.8 kg/m^2^, 1 year after MBS, it was 28.6 kg/m^2^, and before BCS, it was also 28.6 kg/m^2^, indicating BMI stabilization after the bariatric procedure and before proceeding to BCS. The reported associated health problems were hypertension, dyslipidemia, type 2 diabetes mellitus, and anemia. Regarding COVID-19, 91.7% of the responders had contracted the disease at least once. Seventy-five percent of the responders were active smokers, whereas 54.2% reported alcohol consumption socially. Further analysis showed that the two groups were comparable in their demographic data (age, sex, area of residence, marital status, BMIs at different time points, associated health problems, COVID-19 status, smoking status, and alcohol consumption) and did not present statistically significant differences between them (Table 2).

**Figure 2:**
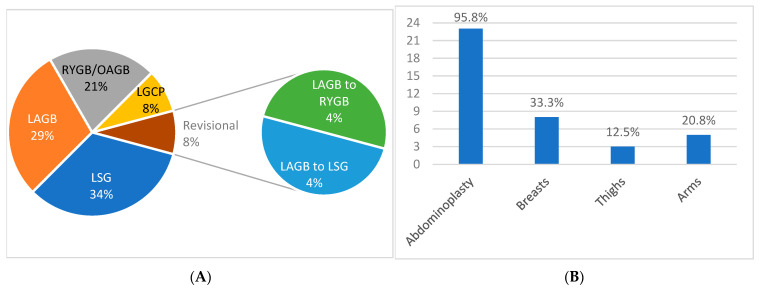
(**A**). Distribution of metabolic bariatric operations in the entire cohort of 24 patients. (**B**). Distribution of body contouring operations in the same cohort. Key: LAGB—laparoscopic adjustable gastric band; LSG—laparoscopic sleeve gastrectomy; LGCP—laparoscopic greater curvature plication; RYGB—Roux-en-Y gastric bypass; OAGB—one-anastomosis gastric bypass.

Regarding the bariatric status of both groups combined (N = 24), 22 patients had undergone one bariatric operation (91.7%) and two patients had undergone two bariatric procedures (8.3%). The distribution of index procedures was as follows: LSG, eight patients (33.3%); LAGB, seven patients (29.2%); RYGB or OAGB, five patients (20.8%), LGCP, two patients (8.3%) (Figure 2A). There was one post-MBS complication documented (one case of leak following LSG). The mean follow-up after the last MBS was 5 years (range: 1–12 years). Regarding the history of BCS in both groups combined, 18 patients (74%) underwent one BCS session, 4 (16.7%) underwent two sessions, and 2 (8.3%) underwent three sessions. Twenty-three patients (95.8%) underwent abdominoplasty, eight (33.3%) underwent breast contouring, three (12.5%) underwent thigh contouring, and five (20.8%) underwent arm contouring (Figure 2B). The mean follow-up after the abdominoplasty was 27 months (range: 1–68 months), and the mean follow-up after the last body contouring operative session was 22 months (range: 1–77 months). The mean interval between the last bariatric operation and the initial body contouring operative session was 2.9 years (range: 6 months–8 years). The mean time elapsed between the abdominoplasty and the interview was 33.9 months, whereas the mean time between the last BCS and the interview was 27.2 months. The overall loss to follow-up was 11.9%; in other words, there was no breach in the follow-up protocol among the patients who participated in this survey. There were no complications documented following BCS, including seroma, hematoma, wound dehiscence, abscess, skin necrosis, deforming scar, or venous thromboembolism.

Regarding the QoL, appearance, and doctor–physician communication component of the survey (Table 1), we divided it into two different sub-questionnaires, one “positive” (17 items: 1–7, 10, 15–18, 20, 21–25) and one “negative” (8 items: 8, 9, 11–14, 19, 22). For each sub-questionnaire, we calculated the total score (i.e., the sum of all items for all patients in each cohort), the crude mean (i.e., the sum of each item divided by the number of patients in each cohort), and the mean-by-item (i.e., the sum of each item divided by the number of patients in each cohort and by the number of items). Regarding the positive sub-questionnaire, the total score for the entire cohort of 24 patients was 333 (out of a potential maximum of 408, should all 24 patients had answered YES to all 17 questions), the respective crude mean was 19.59 (out of 24, should all patients had responded YES), and the mean-by-item was 0.82 (out of 1.00, should all patients had responded YES). Twelve out of 17 positive items achieved a mean by item of ≥0.80. In other words, more than 80% of patients were satisfied with 12/17 positive components (70.6%). For the Plastic sub-cohort, the total score was 266 (out of 323), the crude mean was 15.65, and the mean-by-item was 0.82 (out of 1.00), while the respective figures for the Bariatric sub-cohort were 67 (out of 85), 3.94 (out of 5), and 0.79 (out of 1.0). As far as the negative sub-questionnaire is concerned, the total score for the entire cohort was 21, the crude mean was 2.63, and the mean-by-item was 0.11. Only one out of eight negative items had a mean-by-item of >0.20, i.e., only one negative component (12.5%) was acknowledged by >20% of patients. The respective figures for the Plastic sub-cohort were 19, 2.38, and 0.12, and for the Bariatric sub-cohort, they were 2, 0.25, and 0.05. Supplementary Table S1 summarizes these figures for each item separately, along with their respective sums and means. When comparing the mean-by-item of the positive answers between the Plastic (0.82 ± 0.14) and the Bariatric (0.79 ± 0.29) cohorts, there was no statistically significant difference (*p* = 0.648). The same was true regarding the negative sub-questionnaire (Plastic 0.12 ± 0.17, Bariatric 0.05 ± 0.09, *p* = 0.290).

**Figure 3:**
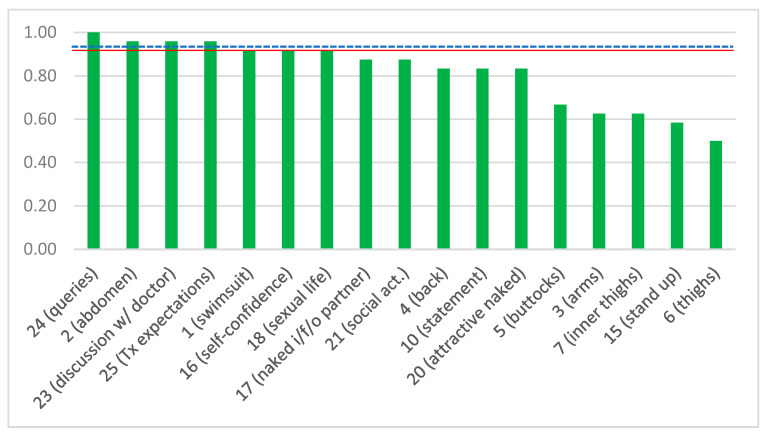
Degree of satisfaction by positive item in descending order. The numbers represent items in the questionnaire, and in the parentheses, there is a brief description of each item. The *y*-axis represents the mean score by item. A threshold of ≥0.80 was considered indicative of satisfaction (red continuous line). The blue dotted line indicates the entire cohort mean by positive item (0.82).

**Figure 4:**
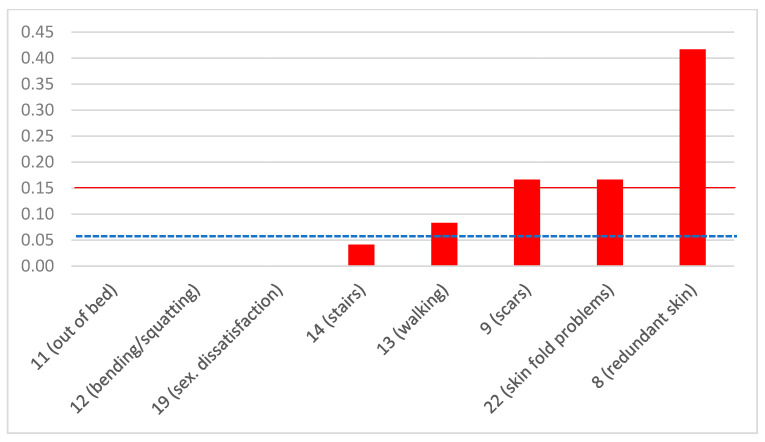
Degree of dissatisfaction by negative item in ascending order. The numbers represent items in the questionnaire, and in the parentheses, there is a brief description of each item. The *y*-axis represents the mean score by item. A threshold of ≥0.20 was considered indicative of dissatisfaction (red continuous line). The blue dotted line indicates the entire cohort mean by negative item (0.11).

The answers with a mean-by-item of <0.80, i.e., the items that the patients reported to be less satisfied with, were item 3 (satisfied with how clothes are shaped by arms; 0.63), item 5 (satisfied with appearance of buttocks; 0.67), item 6 (satisfied with shape of thighs; 0.50), item 7 (satisfaction with appearance of inner surface of thighs; 0.63), and item 15 (standing up for a long time; 0.58). Figure 3 summarizes the mean-by-item for the positive sub-questionnaire in descending order. Among the negative answers, the only one that received a mean ≥0.20 was item 8 (how others look at redundant skin; 0.42). Figure 4 summarizes the mean by item for the negative sub-questionnaire in ascending order.

**Figure 5:**
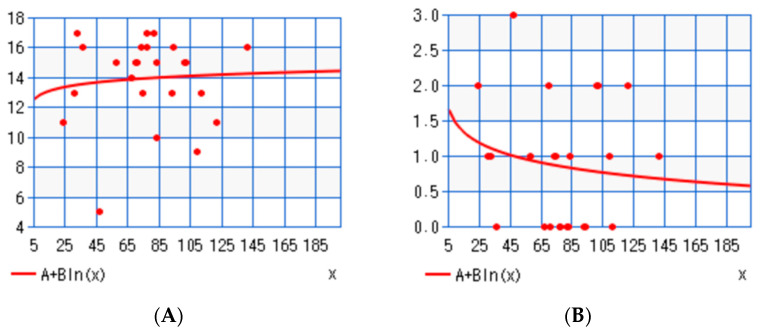
(**A**). Correlation of %EBMIL with positive answers in the survey questionnaire. (**B**). Correlation of % EBMIL with negative answers. The slightly positive (but non-significant) correlation of %EBMIL with positive findings and its slightly negative (but non-significant) correlation with negative findings may be indicative of the fact that patients who have a favorable bariatric outcome are overall satisfied with their body image following body contouring. Key: %EBMIL—percentage of excess of BMI lost. Red dots represent collected data and red lines represent trend of correlation according to this data.

Finally, we attempted to correlate the %EBMIL (percentage of excess of BMI lost) of each patient, a surrogate marker of bariatric effectiveness, with the sum of positive and the sum negative answers that each patient gave, by means of simple linear regression. The time-points for %EBMIL were set before the first bariatric operation and before the first body contouring operation. According to the Shapiro-Wilk *p*-value (0.0053 for positive answers and 0.0041 for negative answers), our data did not follow a normal distribution. As such, we implemented logarithmic linear regression to overcome non-normality. There was no correlation between %EBMIL and either positive answers (*r* = 0.08) or negative answers (*r* = −0.14), but there was a positive and a negative trend, respectively. Figure 5A,B graphically depicts the correlation of %EBMIL with positive and negative findings, respectively.

## 4. Discussion

With the present study, we attempted to capture the current status of body contouring following metabolic bariatric surgery in Greece, according to real-world data from one of the country’s highest-volume tertiary hospitals. In the past, there had been a publication on the desire on behalf of bariatric patients for body contouring following LSG [22]. To our knowledge, the study in hand is the first comprehensive report from Greece on all types of MBS regarding patients who were eventually submitted to BCS.

According to our study, the most striking conclusion is that the prevalence of body contouring after metabolic bariatric surgery is suboptimal, with only 5 out of 229 patients (2.5%) in the Bariatric cohort eventually undergoing BCS. This percentage is even lower than that in previous reports not only from Greece (3.6%) [22], but also from the international literature [9,17]. The most common reason in our cohort was age >50. There is a misconception among healthcare providers and patients alike that BCS after MBS is an aesthetic intervention that suits only younger patients. On the contrary, the typical post-bariatric patient is a middle-aged female, who could potentially benefit further from BCS on the grounds of bariatric outcome, quality of life, and general well-being, as discussed in the Introduction [23]. Therefore, health-care providers, and especially bariatric surgeons, should encourage their patients to undergo BCS once their BMI has stabilized, provided they satisfy the established selection criteria. Furthermore, plastic and reconstructive surgeons should assume a more proactive role when consulting in the context of the multi-disciplinary bariatric team. The second most common reason was inability to cover the costs or non-reimbursement by insurance companies to support this direction, which is a recognized issue worldwide [17,23]. Healthcare providers and policymakers should accumulate the evidence on the benefits of BCS for overall health, with the aim of adjusting the prevailing reimbursement policies. Finally, it is the duty of the healthcare provider to raise awareness on an individual patient basis, underlining that bariatric surgery is not the end of the road in the fight against obesity, but a pivotal step among several to follow, among which body contouring is an integral part.

Another reason that may have led to a decline in BCS in recent years is the adverse impact that the COVID-19 pandemic has imposed on elective surgeries. Although many countries have adjusted their protocols and redirected their caseloads [24], the strike is evident worldwide regarding general surgical practice [25], as well as bariatric and plastic surgery in particular [26,27]. In addition, it has been shown on many occasions that not only was MBS safe during the COVID-19 pandemic, but, first and foremost, it may have a protective effect on a high-risk population, such as patients living with obesity [28,29]. As such, reluctance to perform or undergo either MBS or BCS in view of the COVID-19 sequelae cannot be justified.

In our study, both bariatric operations and body contouring proved to be safe, as there were no immediate and short-term postoperative complications documented except for one case of leak following MBS. This conforms with international reports and constitutes another important message for both bariatric and body contouring candidates [30,31,32,33].

One of the most important components of our study was the satisfaction of our patients regarding their body image and their QoL following BCS, as it was documented through their answers in the provided questionnaire. The majority of positive components (70.6%) regarding body image, social life, sexual life, or doctor–patient communication were appraised by >80% of the participants. Conversely, only 12.5% of negative components were endorsed by >20% of patients. Overall, the participants from both sub-cohorts declared a satisfactory appearance and quality of life, as represented in our questionnaire, in accordance with international literature [15,16,17]. As has been stressed in a recent seminal comprehensive review by Sadeghi et al., abdominoplasty (and BCS in general) significantly improves health-related quality of life (HRQoL) after bariatric surgery [34]. Most importantly, BCS may contribute either directly (through molecular and metabolic effects on adipose tissue) or indirectly (via HRQoL amelioration and improvements in bodily satisfaction) to decreased weight regain after MBS. Taken together, all associated healthcare providers should put greater effort to incorporate BCS in the management of the post-MBS patient as part of an integrated management scheme.

Our study has specific limitations that deserve special mention. To begin with, the small sample size prevents the generalization of conclusions. However, the goal of the survey was to pave the way for larger-scale studies on a national level, with the co-ordination of diverse departments from different cities. Second, the short follow-up period involving certain patients who underwent BCS, especially from Group 2, must be acknowledged, especially with regards to medium- and long-term complications. Nevertheless, short-term complications should not be an issue, given that all included patients had a follow-up course of more than 1 month. Another limitation is that a considerable proportion of patients had undergone gastric banding as the initial and only bariatric operation, a procedure that tends to be abandoned because of its complications in the long run and its suboptimal long-term bariatric outcomes [35,36]. Still, it must be acknowledged that, not infrequently, the bariatric surgeon comes across gastric bands in the context of revisional surgery. Future studies should focus on the most effective and prevalent metabolic bariatric procedures. Perhaps the most critical restraint of our study is the implementation of a non-standardized questionnaire rather than an established one as the BODY-Q, for example [18]. This is a serious restriction with regards to the external validation of our study, generalizability of the results to other populations, and comparisons of the outcomes to those of similar studies. Nevertheless, we made efforts to keep the number of items down to a minimum and render the questionnaire generally concise. In addition, according to our previous empirical and anecdotal experience, patients are more likely to answer a YES/NO type of questionnaire rather than one based on a Likert-scale. Having this in mind, in the questionnaire implemented in this study, we attempted to simplify responses in order to minimize time needed to complete it and consequently maximize patient participation. Undoubtedly, a larger scale study should include a standardized and validated questionnaire.

In conclusion, our single-center study documented a low penetration of body contouring after metabolic bariatric surgery in our country, following a general trend observed on a global scale. Nevertheless, BCS has a clear benefit on quality of life, body image, social life, and sexual life and may even contribute to further and sustainable weight loss, regardless of patient age or the type of bariatric operation. Patient education, adherence to follow-up, and integration of plastic surgeons in the multi-disciplinary bariatric team could potentially attenuate discrepancies in the field. Larger-scale studies are required to yield more solid conclusions and establish correlations between the bariatric outcome and patient satisfaction indices.

## Figures and Tables

**Table 1:** The questionnaire that we utilized for the purposes of our study regarding the quality of life (QoL) following body contouring surgery after metabolic bariatric surgery.

Item No.	Question	Answer	Type of Question
1	Are you satisfied with how your body looks when you wear a swimsuit?	YES/NO	Positive
2	Are you satisfied with how your clothes fit your belly?	YES/NO	Positive
3	Are you satisfied with how your clothes are shaped by your arms?	YES/NO	Positive
4	Are you satisfied with your back looks when you are naked?	YES/NO	Positive
5	Are you satisfied with how your buttocks appear?	YES/NO	Positive
6	Are you satisfied with the shape of your thighs?	YES/NO	Positive
7	Are you satisfied with the looks of the inner surface of your thighs?	YES/NO	Positive
8	Are you annoyed by how others look at your redundant skin?	YES/NO	Negative
9	Are you annoyed by how visible your scars are?	YES/NO	Negative
10	Do you agree with the statement: “my body is not perfect, but I like it”?	YES/NO	Positive
11	Do you have difficulty getting out of bed?	YES/NO	Negative
12	Do you have difficulty bending or squatting (i.e., to tie your laces)?	YES/NO	Negative
13	Do you have difficulty walking?	YES/NO	Negative
14	Do you have difficulty climbing stairs?	YES/NO	Negative
15	Can you stand up for a long time?	YES/NO	Positive
16	Are you self-confident?	YES/NO	Positive
17	Do you feel comfortable when you are naked in front of your partner?	YES/NO	Positive
18	Are you satisfied with your sexual life?	YES/NO	Positive
19	If the answer to the previous question is NO, do you consider that your appearance after the bariatric operation is responsible?	YES/NO	Negative
20	Do you feel attractive when naked?	YES/NO	Positive
21	Do you participate in social activities?	YES/NO	Positive
22	Do you have irritation, rash, or itching in any skin fold of yours?	YES/NO	Negative
23	Do you think that your doctor had a comprehensive discussion with you?	YES/NO	Positive
24	Did your doctor answer all your queries regarding your BCS?	YES/NO	Positive
25	Do you feel that your doctor treated you according to your expectations?	YES/NO	Positive

Key: BCS—body contouring surgery.

**Table 2:** Demographic data of the studied groups of patients who underwent body contouring after metabolic bariatric surgery.

Characteristic	Both Groups N = 24 ±SD, (%)	Group 1 N = 5 ±SD, (%)	Group 2 N = 19 ±SD, (%)	*p*
Age (years)	41.2 ± 7.35	40.2 ± 9.96	41.5 ± 7.04	0.889 (U = 45)
Gender (female)	17 (70.8)	4 (80.0)	13 (68.4)	1.000
Residence (Athens)	17 (70.8)	4 (80.0)	13 (68.4)	1.000
Marital status				
Single	6 (25)	2 (40.0)	4 (21.1)	0.568
Married	16 (66.7)	2 (40.0)	14 (72.7)	0.289
Divorced	2 (16.7)	1 (20.0)	1 (5.3)	0.380
BMI				
Before MBS (kg/m^2^)	43.8 ± 9.86	46.9 ± 7.02	42.2 ± 10.48	0.412 (*U* = 35.5)
1 year post-MBS (kg/m^2^)	28.6 ± 4.25	26.9 ± 6.68	29.1 ± 3.63	0.569 (*U* = 39)
Before BCS (kg/m^2^)	28.6 ± 4.27	26.5 ± 5.00	29.3 ± 4.13	0.201 (*U* = 29)
Maximum (kg/m^2^)	44.3 ± 10.96	49.0 ± 8.88	43.1 ± 11.61	0.242 (*U* = 30.5)
Minimum (kg/m^2^)	25.6 ± 3.40	24.8 ± 5.97	25.8 ± 2.70	0.254 (*U* = 31)
At interview (kg/m^2^)	28.0 ± 4.71	27.0 ± 7.66	28.3 ± 4.02	0.284 (*U* = 32)
Associated health problems				
Hypertension	8 (33)	2 (40.0)	6 (31.6)	1.000
Dyslipidemia	3 (12.5)	0 (0.0)	3 (15.8)	1.000
Type 2 diabetes mellitus	1 (4.2)	0 (0.0)	1 (5.3)	1.000
Anemia	3 (12.5)	2 (40.0)	1 (5.3)	0.099
COVID-19	22 (91.7)	4 (80.0)	18 (94.7)	0.380
Smoking	18 (75.0)	3 (60.0)	15 (78.9)	0.568
Alcohol (social consumption)	13 (54.2)	3 (60.0)	10 (52.6)	1.000

Key: N—sample size; SD—standard deviation; MBS—metabolic bariatric surgery; BCS—body contouring surgery.

## Data Availability

Data are unavailable due to privacy and ethical restrictions.

## Supplementary Materials

The following supporting information can be downloaded at: https://www.mdpi.com/article/10.3390/jcm12093196/s1. File S1: The entire questionnaire as it was distributed to the participants in the Greek language; Table S1: Results, sums, and means of the Quality-of-Life questionnaire.
