# Supplementary material for: Status of Body Contouring Following Metabolic Bariatric Surgery in a Tertiary Hospital of Greece—Still a Long Way to Go
Source: J Clin Med. 2023 Apr 29;12(9):3196. doi: 10.3390/jcm12093196 (PMC10179396; doi:10.3390/jcm12093196)
Supplement: Supplementary file 1 [file jcm-12-03196-s001.zip › File S1.pdf]

Body contouring following bariatric metabolic surgery in a high-volume tertiary centre of Greece: a cross-sectional study on safety, effectiveness, and quality of life.

# - ΕΡΩΤΗΜΑΤΟΛΟΓΙΟ -

ΕΝΟΤΗΤΑ 1 - ΔΗΜΟΓΡΑΦΙΚΑ ΣΤΟΙΧΕΙΑ

Φύλο: Α □, Γ □, Ημ/νία γέννησης: …… / …… / ……….., Ύψος (m): ……,

Επάγγελμα: …………….., Εκπ/ση: α'βάθμια □, β'βάθμια □, γ'βάθμια □, MSc □, PhD □.

Τόπος καταγωγής: …………………………, Τόπος διαμονής: …………………………,

Οικογενειακή κατάσταση: άγαμος □, έγγαμος □, διαζευγμένος □, χήρος/α □.

	Βάρος (Kg)	BMI (Kg/m^2)
**Πριν από την πλαστική επέμβαση**		
**Μέγιστο βάρος ως ενήλικας**		
**Ελάχιστο βάρος ως ενήλικας**		
**Κατά τη συνέντευξη**		

ΕΝΟΤΗΤΑ 2 - ΑΤΟΜΙΚΟ ΑΝΑΜΝΗΣΤΙΚΟ

□ ΑΥ
□ ΔΛΔ
□ ΣΔτ2
□ ΣΝ/ΟΕΜ
□ ΣΚΑ
□ ΑΕΕ
□ ΧΑΠ
□ ΧΝΝ
□ αγγειΐτιδα
□ ΣΕΛ
□ ΡΑ
□ ΙΦΝΕ
□ πεπτικό έλκος
□ αναιμία

□ νόσηση Covid
φορές: ____________

□ κάπνισμα
pack-years: _________

□ αλκοόλ
κοινωνικά ή όχι: ______

ΑΛΛΟ: ______________ ______________________

______________________ ______________________

______________________ ______________________

ΧΦΑ: __________________

____

____

____

ΧΑ: ____________________

ΕΝΟΤΗΤΑ 3 - ΑΣΦΑΛΕΙΑ & ΑΠΟΤΕΛΕΣΜΑΤΙΚΟΤΗΤΑ

Υποβληθήκατε σε <u>βαριατρική</u> επέμβαση; ΝΑΙ □, ΟΧΙ □.

- Αν ΝΑΙ, ημερομηνία βαριατρικής επέμβασης: …… / …… / ………… .

- Είδος επέμβασης: Δακτύλιος □, Πτύχωση □, Sleeve □, Bypass □, ΑΛΛΟ: _______.

- ΜΤΧ επιπλοκές: διαφυγή □, αιμορραγία □, εν τω βάθει φλεβοθρόμβωση □, πνευμονική εμβολή □, θρόμβωση σπληνικής φλέβας □, οξεία νεφρική βλάβη □, πνευμονία □, οξύ στεφανιαίο επεισόδιο □, εσωτερική κήλη □, χολοκυστίτιδα □, εμφάνιση ΓΟΠ □, αναστομωτικό έλκος □, ΑΛΛΟ: ___________________________.

- Βάρος πριν από τη βαριατρική επέμβαση: ________ Kg.
- Βάρος 6 μήνες μετά τη βαριατρική επέμβαση: ________ Kg.
- Βάρος 1 έτος μετά τη βαριατρική επέμβαση: _________ Kg.
- Βάρος 2 έτη μετά τη βαριατρική επέμβαση: __________ Kg.

Υποβληθήκατε σε <u>πλαστική</u> επέμβαση; ΝΑΙ □, ΟΧΙ □.

- Αν ΝΑΙ, ημερομηνία πλαστικής επέμβασης: …… / …… / ………… .

- Είδος επέμβασης: Κοιλιοπλαστική □, Μαστοί □, Γλουτοί □, Βραχίονες □, ΑΛΛΟ: ____.

- ΜΤΧ επιπλοκές: σέρωμα □, αιμάτωμα □, διάσπαση τραύματος □, διαπύση/απόστημα □, δερματική νέκρωση □, παραμορφωτική ουλή □, εν τω βάθει φλεβοθρόμβωση □, πνευμονική εμβολή □, ΑΛΛΟ: _________________________.

ΕΝΟΤΗΤΑ 4 - ΠΟΙΟΤΗΤΑ ΖΩΗΣ

	ΝΑΙ	ΟΧΙ
Είστε ικανοποιημένος/η με το πώς δείχνει το σώμα σας με μαγιό;		
Είστε ικανοποιημένος/η με το πώς μπαίνου τα ρούχα στην κοιλιά;		
Είστε ικανοποιημένος/η με το πώς διαγράφονται τα μπράτσα στα ρούχα;		
Είστε ικανοποιημένος/η με το πώς δείχνει η πλάτη σας γυμνή;		
Είστε ικανοποιημένος/η με το μέγεθος των γλουτών σας;		
Είστε ικανοποιημένος/η με το σχήμα των μηρών σας;		
Είστε ικανοποιημενο/η με το πώς δείχνει το δέρμα στους λαγόνες σας;		

	ΝΑΙ	ΟΧΙ
Σας ενοχλεί το πώς βλέπουν οι άλλοι το πλεονάζον δέρμα σας;		
Σας ενοχλεί το πόσο ευδιάκριτες είναι οι ουλές σας;		
Συμφωνείτε με την ακόλουθη δήλωση: “το σώμα μου δεν είναι τέλειο αλλά μου αρέσει”;		
Δυσκολεύεστε να σηκωθείτε από το κρεβάτι;		
Δυσκολεύεστε στο σκύψιμο (π.χ. γα να δέσετε τα κορδόνια σας);		
Δυσκολεύεστε στο περπάτημα;		
Δυσκολεύεστε όταν ανεβαίνετε σκάλες;		
Αντέχετε την ορθοστασία για αρκετή ώρα;		
Έχετε αυτοπεποίθηση;		
Αισθάνεστε άνετα όταν γδύνεστε μπροστά στον/στην σύντροφό σας;		
Είστε ικανοποιημένος/η από τη σεξουαλική σας ζωή;		
Αισθάνεστε ελκυστικός/η όταν είστε γυμνός/η;		
Συμμετέχετε σε κοινωνικές δραστηριότητες;		
Έχετε ερεθισμό, εξάνθημα, ή φαγούρα σε δερματικές πτυχές;		
Θεωρείτε ότι ο γιατρός σας σας μίλησε με κατανοητό τρόπο;		
Απάντησε ο γιατρός σας σε όλες τις απορίες σας σχετικά με την επέμβαση;		
Αισθάνεστε ότι ο γιατρός σας αντιμετπώισε με σεβασμό;		

—————————— ΠΕΡΑΣ ΕΡΩΤΗΜΑΤΟΛΟΓΙΟΥ ——————————
